# Aging Society and Environmental Health Challenges

**DOI:** 10.1289/ehp.1206334

**Published:** 2013-03-01

**Authors:** Yun-Chul Hong

**Affiliations:** Institute of Environmental Medicine, Department of Preventive Medicine, Seoul National University College of Medicine, Seoul, Korea, Email: ychong1@snu.ac.kr

The world population is aging. In fact, between 2000 and 2050 the global population ≥ 60 years of age is projected to more than triple, reaching nearly 2 billion, and the elderly population is growing faster than the total population in practically all regions of the world (United Nations 2001).

Recently, aging has received increasing attention. The theme of World Health Day in 2012 was aging and health. In addition, at a gerontology congress held in Cuba in March 2012, Margaret Chan stated that “within the next five years, for the first time in history, the number of adults aged 65 and older will outnumber children under the age of 5” (Chan 2012).

However, living longer does not necessarily mean having a better or healthier life. With advancing age of the population, the prevalence of age-related diseases tends to increase dramatically. Thus, to reduce the burden of age-related diseases, it is important to identify and avoid potential risk factors for diseases such as cancer, diabetes mellitus, and cardiovascular disorders. Among the many factors that affect the aging process, environmental exposure is one of the modifiable risk factors. Therefore, we need to better understand the risks to the elderly from environmental exposure. Environmental pollutants and chemicals adversely influence the homeostatic status of aging, frequently resulting in development of certain diseases at an earlier age than expected.

This phenomenon was unknown in the past because people died as a result, for example, of illness, injury, or hostility before reaching old age (Santangelo et al. 2011). However, the life span has increased substantially, up to the end stage of our biological programming, mostly due to improved living conditions. The question now is how environmental conditions (e.g., contaminants, climate change) are detrimental to the health of the elderly.

Ironically, environmental contaminants, most from anthropogenic sources originally intended to improve living conditions, are damaging human health, particularly in susceptible populations. The elderly are more sensitive because of deterioration in physiologic, biochemical, immunologic, and homeostatic parameters, which affects their ability to defend against environmental stresses. Xenobiotic metabolism also reduces with age, resulting in increases in toxic metabolites. Any compromised function of organ systems may result in the reduced ability to protect the body from adverse effects of xenobiotics (Risher et al. 2010). Increased oxidative stress from environmental exposures may also contribute to initiation and progression of cardiovascular and pulmonary diseases. Aging itself has been associated with increased oxidative stress and lower antioxidant defense (Bokov et al. 2004). In addition, the elderly have likely been exposed to toxicants for long periods of time because of their age.

A few epidemiologic studies have investigated the adverse effects of environmental exposures on age-related diseases. One of these is the Normative Aging Study, an investigation of aging established in 1963 by the U.S. Veterans Administration. Using this cohort, Park et al. (2006) suggested that “elderly men with [metabolic syndrome] were more susceptible to autonomic dysfunction in association with chronic lead exposure.” In a study examining whether the combined effects of lead and stress modified the relationship between age and cognitive impairment, Peters et al. (2010) suggested that cognitive impairment was not a result of aging alone but resulted from the combined effects of risk factors, such as lead exposure, and age. Recently, Schwartz et al. (2012) reported that long-term exposure to traffic particles was associated with increased blood pressure, an established risk factor for coronary heart disease and stroke in elderly men. The Normative Aging Study has greatly contributed to our understanding of interactions among aging, development or aggravation of diseases, and environmental exposure, particularly low levels.

In the Korean Elderly Environmental Panel (KEEP) study, a study with a repeated-measure design, short-term exposure to air pollution was significantly associated with elevated insulin resistance, which is regarded as a core pathogenetic mechanism of diabetes mellitus or metabolic syndrome (Kim and Hong 2012). In another study using the KEEP cohort, Bae et al. (2012) reported that bisphenol A exposure was associated with increased blood pressure and decreased heart rate variability, suggesting that the elderly might be more susceptibile to the possible cardiovascular effects of this ubiquitous endocrine-disrupting agent.

In addition to vulnerability to environmental pollutants, the elderly are also vulnerable to the health impacts of climate change, such as heat waves and extreme weather events (Frumkin et al. 2012). Recent studies have consistently reported a strong relationship between heat and heat waves and increasing death rates among the elderly, particularly for respiratory and cardiovascular mortality (reviewed by Åström et al. 2011).

Study of environmental health in the elderly has a great deal in common with the study of children’s health because it emphasizes the dynamic interplay of timing, functions, and environmental exposures. Obviously, the major contrast of these two types of study is declining functions over time in the elderly compared with growth and development in children. Children are not just smaller adults, nor are the elderly just older adults; they are individuals with unique challenges and medical needs different from those of younger adults (Risher et al. 2010). Because of the variability of health effects resulting from exposure to environmental agents in the elderly, individual functions should be viewed in the context of genetics, health status, nutrition, occupation, and socioeconomic status. In terms of environmental exposure, the elderly are expected to have cumulative insults from past exposure, but with current community levels likely to be lower and probably different from the past levels they experienced. The complex interactions among the aging process, sources of variability, and changing exposure levels present remarkable challenges. To tackle these complexities, we need to carefully consider research design, population sample, historical period, measurement, and analyis to ensure validity of study findings. Longitudinal designs, such as cohort or panel studies, are probably necessary for better understanding these interactions in the context of variability. The challenge will be using data regarding behavior/activity patterns, exposure to pollutants, and the changes in pharmacokinetic and pharmacodynamic factors in the elderly (Geller and Zenick 2005). International collaborative studies will be necessary to assess the reproducibility and generalizability of findings.

In her speech, Chan (2012) noted that “being in the older age group is becoming the ‘new normal’ for the world’s population. Our ‘seniors’ are now our biggest age group.” This means that this population that is potentially more susceptible to environmental contaminants is becoming larger. Therefore, we need to adapt environmental health policies to address these future challenges. It will be difficult to reduce or prevent environmental factors from aggravating age-related illness, and these illnesses and how to address them will become more serious in the future.

## References

[r1] Åström DO, Forsberg B, Rocklöv J (2011). Heat wave impact on morbidity and mortality in the elderly population: a review of recent studies.. Maturitas.

[r2] Bae S, Kim JH, Lim YH, Park HY, Hong YC (2012). Associations of bisphenol A exposure with heart rate variability and blood pressure.. Hypertension.

[r3] Bokov A, Chaudhuri A, Richardson A. (2004). The role of oxidative damage and stress in aging.. Mech Ageing Dev.

[r4] Chan M. 2012. The New Normal: Life after Sixty. Lecture Delivered at the Congress on Gerontology and Geriatrics and the 20th International Seminar on Care for the Elderly, Havana, Cuba, 30 March 2012. Available: http://www.who.int/dg/speeches/2012/ageing_20120330/en/index.html [accessed 7 February 2013].

[r5] Frumkin H, Fried L, Moody R. (2012). Aging, climate change, and legacy thinking.. Am J Public Health.

[r6] Geller AM, Zenick H (2005). Aging and the environment: a research framework.. Environ Health Perspect.

[r7] Kim JH, Hong YC (2012). *GSTM1, GSTT1*, and *GSTP1* polymorphisms and associations between air pollutants and markers of insulin resistance in elderly Koreans.. Environ Health Perspect.

[r8] Park SK, Schwartz J, Weisskopf M, Sparrow D, Vokonas PS, Wright RO (2006). Low-level lead exposure, metabolic syndrome, and heart rate variability: the VA Normative Aging Study.. Environ Health Perspect.

[r9] Peters JL, Weisskopf MG, Spiro A, Schwartz J, Sparrow D, Nie H (2010). Interaction of stress, lead burden, and age on cognition in older men: the VA Normative Aging Study.. Environ Health Perspect.

[r10] Risher JF, Todd GD, Meyer D, Zunker CL (2010). The elderly as a sensitive population in environmental exposures: making the case.. Rev Environ Contam Toxicol.

[r11] Santangelo A, Albani S, Beretta M, Cappello A, Mamazza G, Pavano S (2011). Aging and environmental factors: an estimation of the health state of the elderly population residing in industrialized vs. rural areas.. Arch Gerontol Geriatr.

[r12] Schwartz J, Alexeeff SE, Mordukhovich I, Gryparis A, Vokonas P, Suh H (2012). Association between long-term exposure to traffic particles and blood pressure in the Veterans Administration Normative Aging Study.. Occup Environ Med.

[r13] United Nations. 2001. World Population Ageing: 1950–2050. Available: http://www.un.org/esa/population/publications/worldageing19502050/ [accessed 7 February 2013].

